# Measuring Ocular Aberrations Sequentially Using a Digital Micromirror Device

**DOI:** 10.3390/mi10020117

**Published:** 2019-02-12

**Authors:** Alessandra Carmichael Martins, Brian Vohnsen

**Affiliations:** Advanced Optical Imaging Group, School of Physics, University College Dublin, Dublin D04, Ireland; brian.vohnsen@ucd.ie

**Keywords:** wavefront sensing, digital micromirror device, ocular aberrations

## Abstract

The Hartmann–Shack wavefront sensor is widely used to measure aberrations in both astronomy and ophthalmology. Yet, the dynamic range of the sensor is limited by cross-talk between adjacent lenslets. In this study, we explore ocular aberration measurements with a recently-proposed variant of the sensor that makes use of a digital micromirror device for sequential aperture scanning of the pupil, thereby avoiding the use of a lenslet array. We report on results with the sensor using two different detectors, a lateral position sensor and a charge-coupled device (CCD) scientific camera, and explore the pros and cons of both. Wavefront measurements of a highly aberrated artificial eye and of five real eyes, including a highly myopic subject, are demonstrated, and the role of pupil sampling density, CCD pixel binning, and scanning speed are explored. We find that the lateral position sensor is mostly suited for high-power applications, whereas the CCD camera with pixel binning performs consistently well both with the artificial eye and for real-eye measurements, and can outperform a commonly-used wavefront sensor with highly aberrated wavefronts.

## 1. Introduction

Quantification of aberrations is important in a number of applications and needed to optimize performance as, for example, with adaptive optics. One area of special relevance is ophthalmology, where the optical quality of the human eye is important not only for acute vision but also for diagnostic retinal imaging applications.

The optical performance of the human eye is limited not by diffraction but rather by the amount of monochromatic and chromatic aberrations. Ocular wavefront aberrations are mainly induced by the cornea and the crystalline lens and become increasingly important with a larger pupil size. The wavefront aberrations commonly refer to the conjugate pupil plane of the eye, where deviations from a planar wavefront refer to aberrations that prevent light from being focused onto a diffraction-limited spot on the retina. The aberrations for monochromatic light are expressed as a linear combination of orthonormal circular Zernike polynomials weighted by a series of Zernike coefficients, expressed in either μm or wavelength units [1].

Many methods have been proposed since the 1960s to measure aberrations of the human eye both objectively and subjectively including modified aberrometers [2,3], ray tracing techniques [4], and indirectly via retinal images [5]. However, Hartmann–Shack wavefront sensors (HS-WFSs) have become the preferred devices to measure the wavefront and intensity distribution of backscattered light from the retina [6]. The HS-WFS uses a tightly focused beacon of light in the retinal plane, which serves as secondary point source for the wavefront sensing, with a lenslet array that samples the local distribution of wavefront tilt in the pupil plane. Complementary metal-oxide-semiconductor (CMOS)-based HS-WFS can capture aberration changes at 100′s of Hz, although common CCD-based HS-WFS are limited to 10′s of Hz. In either case, cross-talk between adjacent lenslets limit the dynamic range. Modifications to HS-WFS have been proposed to improve the dynamic range by using a liquid-filled lenslet array [7] and by replacing it with a liquid crystal display [8,9], although only few have been tested with ocular aberrations.

As the need to determine ocular aberrations has become increasingly important for personalized refractive corrections using custom LASIK [10], and for the understanding of a number of refractive problems ranging from keratoconus [11] to increased high myopia [12], establishing a wavefront sensing technique that provides a high dynamic range, resolution, and high speed suitable for the human eye is crucial. Digital micromirror devices (DMDs) can operate at speeds in the 10′s of kHz range, while sequential scanning provides a large dynamic range due to the lack of a lenslet array; therefore, avoiding the appearance of cross-talk, as recently proposed by the authors in a DMD-WFS [13]. Additionally, DMDs have recently been used for ophthalmic applications in retinal imaging [14] and psychophysical measurements [15]. A somewhat related technique uses scanning of an incident beam of light in the pupil plane to capture multiple retinal images [16]. Here, the narrow beam of light is incident near the pupil center and scanning is only done for light exiting the eye by sequential aperture scanning of the pupil, analogous to the parallel sampling by a lenslet array in the HS-WFS. Centroiding methods of the imaged point-spread-function (PSF) for ocular aberrations have been compared [17] and can be tuned to provide more accurate wavefront reconstructions in the presence of noise.

Here, the wavefront sensing technique using a DMD is used to measure ocular aberrations of the human eye by performing sequential zonal scanning of the wavefront. The experimental setup and method are explained in Section 2 using two types of detectors. Results are shown in Section 3.1 for an artificial eye and in Section 3.2 for human eyes. A discussion about the results and technique can be found in Section 4, followed by the conclusion of the study in Section 5.

## 2. Materials and Methods

**Figure 1:**
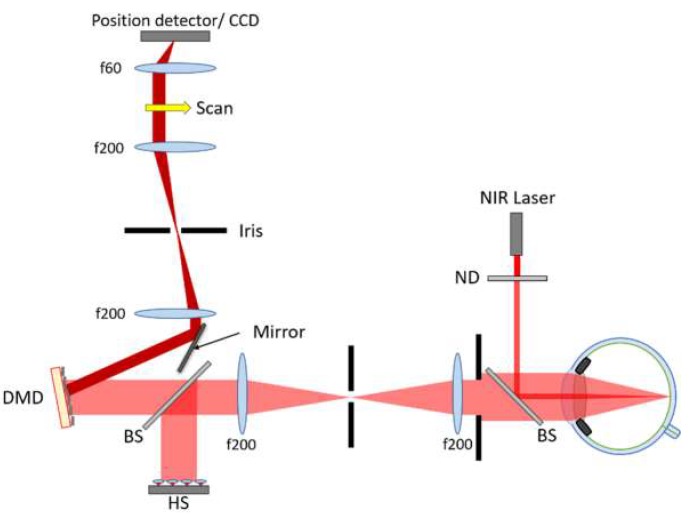
Schematic of the system used to measure ocular aberrations using a digital micromirror devices (DMD). A 4 mm beam of near-infrared (IR) light backscattered from the retina is sequentially scanned in the pupil plane by the DMD, which directs light onto a position detector via a plane mirror to capture the maximum light. The detector captures the (*x*,*y*) centroid coordinates of the point-spread-function (PSF) in the case of the lateral position detector, or captures images of the PSF in case of the charge-coupled device (CCD) camera, for each scanned section of the light. All lenses used are antireflection-coated achromatic doublets.

A wavefront sensor based on sequential scanning of a reflective cell with a DMD (V-7001 VIS, Vialux, Chemnitz, Germany) is used to measure ocular aberrations. The system, which is described in detail in [13], has been adapted for real-time aberration sensing of the human eye. A schematic of the setup can be seen in Figure 1. Essentially, a narrow near-infrared (850 nm) beam of 200 μW entering the eye is focused onto the retina to create a secondary point source suitable for wavefront sensing. Backscattered light exiting the eye is truncated by a 4 mm iris and the pupil is imaged by a 4f telescope onto the DMD. The DMD is comprised of 1024 × 768 mirrored square pixels of 13.7 μm that allow binary positioning at ±12° (and optical angles of ±24°) at up to 22.7 kHz. The DMD divides the imaged pupil into equal-sized cells, consisting of a square array of micromirrors, which are sequentially activated, and the reflected light is focused onto a position detector. Due to diffraction effects from the DMD, an iris is used in a conjugate retinal plane to only allow the pass of the 0th order. In this study, two different position detectors are used:

(1) A 2D-lateral resistive position detector (PDP90A, Thorlabs^TM^, Newton, NJ, USA) of up to 0.75 μm spatial resolution, was used to register the central position of the PSF centroid (*x*,*y*) for each activated DMD cell. Although its angular resolution suffices, the limited sensitivity of this device prevents it from being used to determine ocular aberrations, but it is included here as a proof-of-principle in Section 3.1.1. Indeed, it may well find applications where power limitations are of less concern, such as in the characterization of laser beams.
(2) A CCD camera (Scientific Camera 1501M-USB, Thorlabs^TM^, Newton, NJ, USA) with 6.45 μm pixel pitch and 14-bit digital output is used for the rest of the results in Section 3.1.2 and Section 3.2, allowing for high brightness variations and binning of pixels to increase acquisition speed when acquiring images of the PSF from which the centroid position can be determined.

The DMD, the position detector, and the CCD are all programmed and synchronized via LabVIEW^TM^. Thus, the position detector captures the PSF centroid coordinates at a speed of 1.5 kHz, whereas the CCD captures the PSF images at a speed of 13 frames-per-second (FPS) without pixel binning and 1 ms exposure, but faster with pixel binning of up to 24 × 24 pixels. For example, with 10 × 10 pixel-binning the speed limit is 77 FPS. For both sensors, the aberration is determined with respect to that of a plane reference wave obtained by placing a flat mirror in the pupil plane. A conventional Hartmann–Shack wavefront sensor (HS-WFS 150-5 C, 73 × 73 lenslets, Thorlabs^TM^, Newton, NJ, USA) is placed in a conjugate pupil plane and is used for comparison and verification purposes.

First, a test was performed with an artificial eye comprised of a thin ophthalmic trial lens adjacent to a flat mirror placed in the pupil plane. The DMD-WFS scan was implemented with two sampling densities: (a) 5 × 5 DMD cells of 800 μm (58 × 58 pixels) obtaining the corresponding 25 PSF images, and (b) 10 × 10 DMD cells of 400 μm (29 × 29 pixels) corresponding to 100 PSF images. For each sampling density of the DMD, the PSF images were acquired using 1 × 1, 2 × 2, 4 × 4, and 8 × 8-pixel-binning of the CCD camera, recovering images of 1392 × 1040, 696 × 520, 348 × 260, and 174 × 130 pixels, respectively. A plane reference wave was also attained for each case, and the effects of binning compared.

Secondly, ocular aberration measurements were performed in the right eye of 4 emmetropic subjects (equivalent sphere between 0 and −1 diopters, as measured with an EyeNetra^TM^ autorefractor) and one myopic subject (with an equivalent sphere of −7D) whose pupils were dilated, and accommodation partially paralyzed, with two drops of 1% tropicamide. To ensure a point source on the retina of myopic subjects, a corrective trial lens is placed in a prior conjugate plane to the pupil, making use of a one-to-one 4f system composed of two 150 mm focal length achromatic lenses. A bite bar was used to ensure good centration of the eye’s pupil and reduce head movement throughout the measurements. For their own commodity, subjects were asked to take a short break between one scan measurement and the next, hence slightly readjusting their position for each measurement.

**Figure 2:**
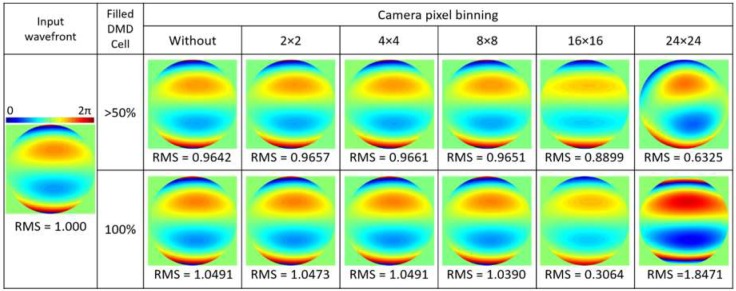
Comparison of wavefront reconstructions of a simulated wavefront with astigmatism, coma, and trefoil. First, using DMD cells which are at least 50% illuminated with a 4 mm beam, and second, those 100% illuminated by the incoming beam. For each case, the effect of square pixel binning of ×2, ×4, ×8, ×16, and ×24 pixels in the detector camera is compared. All root-mean-square (RMS) values are given in μm.

For both cases, the Zernike wavefront parameters obtained from the HS-WFS were recorded up to the 4th radial order. Subsequently, the DMD-WFS scan was performed at 13 FPS, adding up to a total time of 2 s for a complete 5 × 5 sampling and 8 s for a 10 × 10 scan. The PSF images were post-analyzed in Matlab^TM^ to determine the center-of-gravity centroid translations in cartesian coordinates between the aberrated wavefront and that of the reference for each DMD cell. These translations were used to calculate the Zernike coefficients from which the least-square wavefront reconstruction was performed. The procedure of this method is explained in detail in [13]. For 5 × 5 DMD sampling, the PSF corresponding to DMD cells illuminated across more than 50% of their area were analyzed and used for the reconstruction, while for 10 × 10 sampling, only those 100% illuminated were considered. To further examine the effects of using 50% illuminated area versus 100% illuminated area of DMD cells and that of binning, the reconstruction procedure here followed for 5 × 5 DMD cell sampling was compared in Figure 2 for a simulated wavefront with astigmatism, coma, and trefoil, similar to that from [18] for a typical human eye. Reconstructing the wavefront only using the fully illuminated DMD cells, provides more accurate results for a static wavefront. However, when sampling a dynamic wavefront, such as that of the real eye, with a low density and only considering cells illuminated in their entirety, could be insufficient for accurate measurements. On the other hand, increasing pixel binning in the detection camera allows for faster acquisition and is seen to maintain accuracy up to 8 × 8 binned pixels (deviation <4%), after which the accuracy of the wavefront reconstruction tends to fall.

## 3. Experimental Results

Wavefront diagrams and root-mean-square (RMS) wavefront values shown in this section are exempt from tip and tilt, as these are not representative of the ocular aberrations as such, and a minimal decentration is necessary to avoid corneal reflections in the HS-WFS measurements.

### 3.1. Wavefront Aberrations with an Artificial Eye

#### 3.1.1. Lateral Position Detector

**Figure 3:**
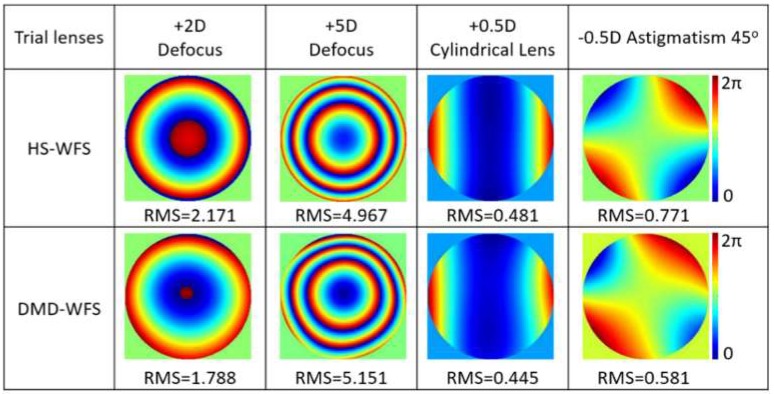
Wavefront reconstructions of an aberrated wave across a 4 mm pupil with ophthalmic trial lenses using the 5 × 5 sampling DMD-WFS method and a 2D-lateral position detector placed in the image plane. All RMS values are given in μm.

The lateral position detector provides the cartesian coordinates of an incident beam with respect to a predetermined center point and is commonly used for system alignment. Here, it was used to determine the centroid coordinates of the PSF and displacements were given with respect to that of a focused plane reference wave. The reconstruction of aberrated wavefronts induced by four different trial lenses can be seen in Figure 3 and compared to the wavefront measured by the HS-WFS. Astigmatism was achieved with two crossed positive and negative power cylindrical lenses. All measurements shown were performed with 5 × 5 DMD cell sampling. Increasing the sampling density to 10 × 10 decreased the amount of optical power per cell that reached the position detector, and; therefore, limited its accuracy with the available power.

#### 3.1.2. CCD Camera Detector

**Figure 4:**
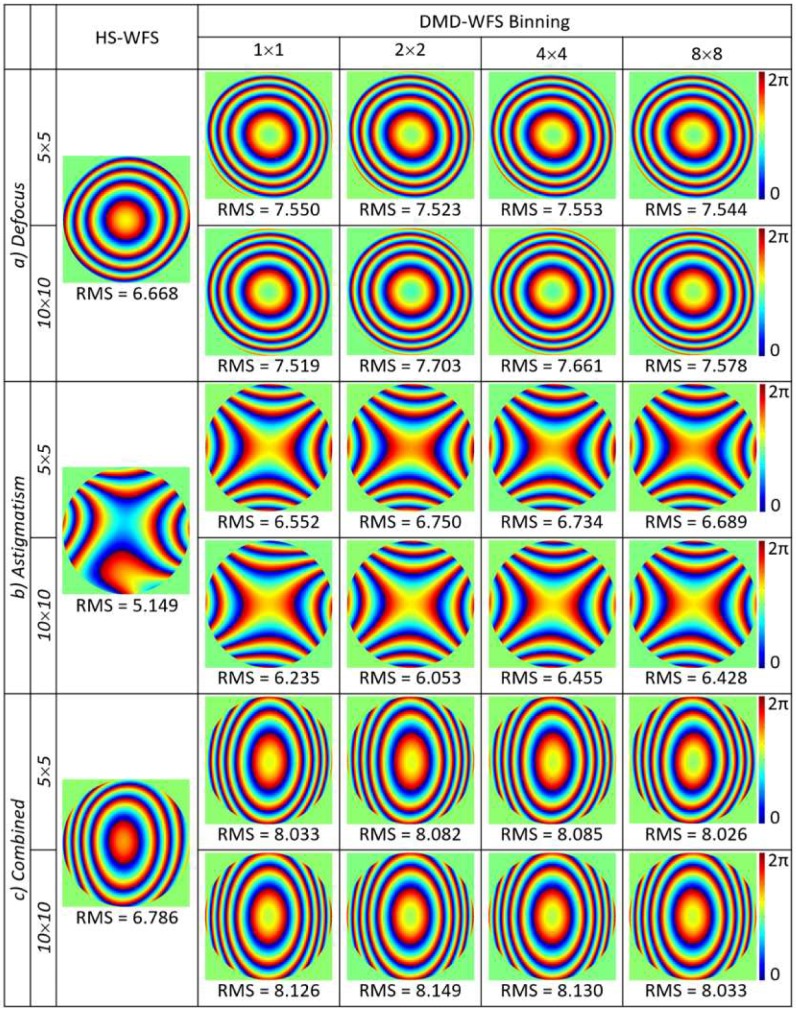
Comparison of defocus and astigmatic wavefront reconstructions for a 4 mm pupil with an artificial eye through Zernike coefficients between HS wavefront sensing, and for the DMD wavefront sensing method with four different binning options in the CCD camera, acquiring the PFS images for each DMD cell. All RMS values of the Zernike coefficients are given in μm.

The wavefront reconstructions of the artificial eye using the Zernike coefficients measured by the HS-WFS and the DMD-WFS were included in Figure 4 for a 4 mm pupil and two sets of ophthalmic lenses: a) defocus with a +8D spherical lens, b) astigmatism with two crossed −4D and +4D cylindrical lenses, and c) combination of defocus and astigmatism with a +8D spherical lens and crossed −4D and +4D cylindrical lenses. Given that the trial lenses were used in conjunction with a flat mirror, the double pass of the beam caused induced aberration to lay beyond the range of the HS-WFS. Different binning options ranging from one to eight pixels were included when measured with the DMD-WFS. The RMS values of the Zernike coefficients were used to quantify and compare the obtained results, with only up to 2.5% deviation for different pixel-binning options. However, a difference of up to 30% was observed when compared to the HS-WFS, where the aberrations were underestimated. The obtained data comparing both methods in terms of Zernike coefficients as well as sphere and cylinder power is detailed in Table 1.

### 3.2. Wavefront Aberrations of the Real Eye

**Figure 5:**
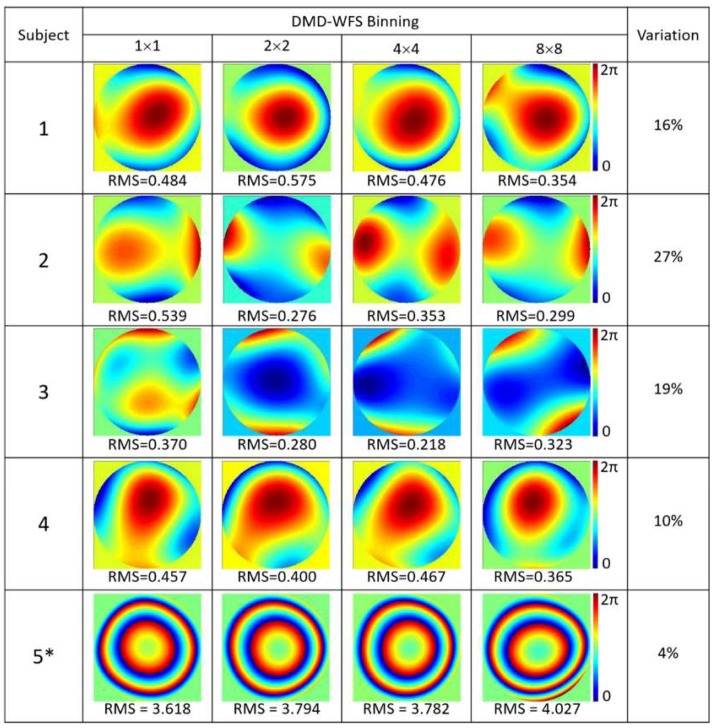
Ocular aberrations measured with the DMD-WFS with 5 × 5 sampling density for four subjects with normal vision and one myopic subject (−7D), marked with an asterisk (*), using DMD cell with at least 50% of its area illuminated. Quantification of wavefronts are presented as RMS values given in μm.

**Figure 6:**
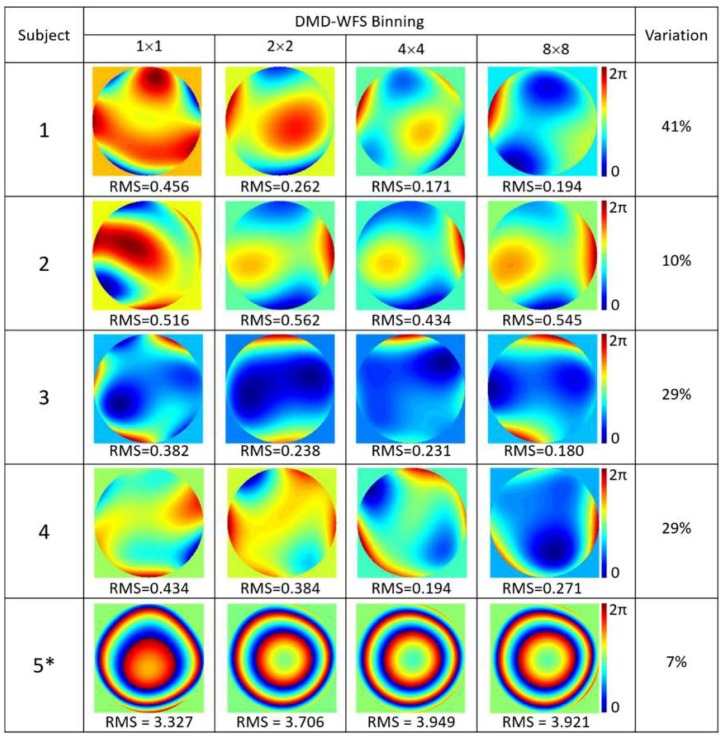
Ocular aberrations measured with the DMD-WFS with 10 × 10 sampling density using only 100% illuminated DMD cells for the same five subjects. The asterisk (*) denotes the myopic subject. All RMS values are given in μm.

Measurements of ocular aberrations of five healthy subjects, four of which were emmetropes and one which was myopic (−7D), are shown in Figure 5 for 5 × 5 sampling density and in Figure 6 for 10 × 10 sampling density. In both cases, four types of pixel binning in the CCD camera were included. Wavefronts were quantified using the RMS value of the Zernike coefficients, given in μm.

Larger variations between the reconstructed wavefronts appeared when measuring the real eye in comparison to the previous results with the artificial eye. This could be due to the natural movement of the eye, which includes both voluntary and involuntary movements, even when fixating on a given target [19]. Fixation time lasted approximately 200–300 ms [20,21], with large variability between subjects, which falls well below the required DMD-WFS scan time. Increasing the pixel binning allowed for a higher acquisition speed to limit variations during ocular aberration measurements. Variations of 5% to 30% in the RMS values were noted between different binning options performed at the same speed for 5 × 5 DMD sampling. Denser sampling of 10 × 10 DMD cells involved higher acquisition time, causing deviations to increase between 7% and 40%.

**Figure 7:**
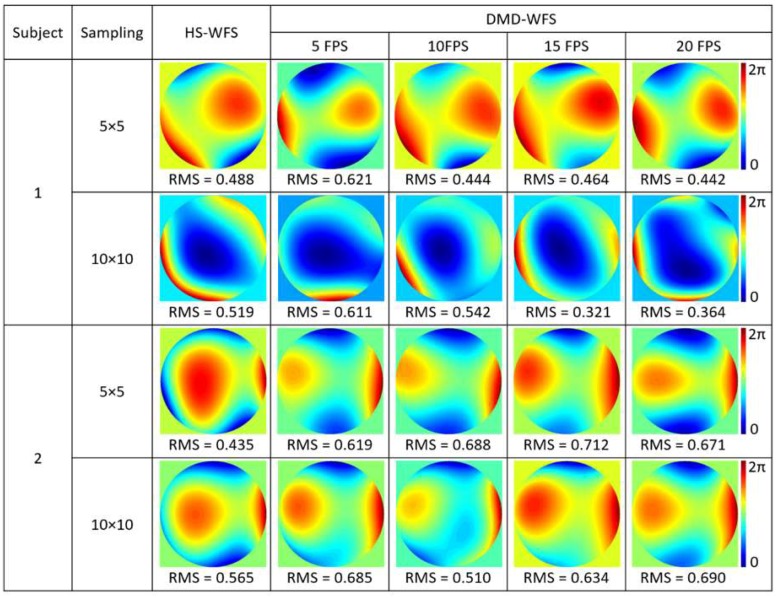
Comparison of the DMD-WFS scan performed at 5, 10, 15 and 20 FPS for two subjects with both 5 × 5 and 10 × 10 DMD cell sampling densities. All RMS values are given in μm.

In order to analyze the effect of scanning at different speeds, measurements were performed in the right eyes of the authors at 5, 10, 15, and 20 frames per second with both 5 × 5 and 10 × 10 DMD cell sampling. Results are included in Figure 7. For each subject, larger differences were seen in the case of 10 × 10 sampling, as the measurement time varied considerably between being performed at 5 FPS or 20 FPS.

## 4. Discussion

The use of a DMD to sequentially scan an aberrated wavefront using a single achromatic lens to focus light onto a position-sensitive detector allows for wavefront measurements with high dynamic range by avoiding crosstalk, which potentially limits the performance of a conventional HS-WFS. This sees applicability in ophthalmology due to the fast-increasing rates of high myopia [22]. Corneal reflections, or undesired corneal areas can also be eliminated by deactivating the corresponding cells in the DMD, such that these do not induce noise into the wavefront reconstruction. An example where reflections can cause problems can be seen in the lower part of Figure 4b for the HS-WFS wavefront with astigmatism.

The use of a position detector device provides direct determination of centroid coordinates with an accuracy of up to 0.75 μm when output voltage is maximized, avoiding the need to save large amounts of image data for each measurement, specifically in the case of large sampling densities and no pixel binning. However, given its high-power requirements for accurate detection, it did not prove feasible for ophthalmic applications.

The CCD camera with a 14-bit optical output working synchronously with the DMD was shown to measure static wavefronts with high precision in the artificial eye. Furthermore, the possibility of binning pixels in *x-* and *y-*directions allow for increased speed and lower amounts of stored data without compromising accuracy. Slight variations in the wavefront RMS values seen in Figure 4 with different binning options of up to 8 × 8 pixels are in the same order of magnitude as predicted by the simulated wavefront from Figure 2.

For ophthalmic applications, the high speed of the DMD and CCD camera to perform a complete scan is crucial. Scans performed at 13 FPS were to suffice when measuring ocular aberrations with both 5 × 5 and 10 × 10 DMD cell sampling, and the effect of scanning speed between 5 and 20 FPS was analyzed. However, variations in the measured wavefronts are still present due the continuous involuntary movements of the eye during fixation including tremors, drifts, and microsaccades [19,20,21], where further increasing the speed would improve accuracy and repeatability. Changes in the tear film, which is known to be dynamic, may also cause changes in the measurements of ocular aberrations [23]. Binning larger amounts of pixels in the detection camera, can help improve the signal; however, compromises accuracy above 8 × 8 pixels, as seen in Figure 2. Additionally, using slightly better centroiding methods [17] could improve accuracy. The use of a high-speed CMOS camera could potentially allow for higher speed, but ultimately a different methodology, such as single-pixel sensing, may be required to gain the upmost in terms of kHz speed [24].

## 5. Conclusions

Sequential scanning of a wavefront using a DMD and a single achromatic lens to measure aberrations, with near-infrared light removing the conventional lenslet array found in HS-WFS, has been achieved. This provides high dynamical range with a trade-off between high sampling densities and speed, where the latter can be increased by pixel binning. This technique grants high adaptability to different applications and is here tested to measure ocular aberrations in artificial and real eyes. Two different wavefront sampling densities were compared, and the effect of pixel binning was analyzed and found to allow for accurate reconstructions for a static artificial eye, but were subject to variations in the real eye, where increased speed would still be paramount for higher accuracy.

## Figures and Tables

**Table 1:** Data comparison between the Zernike coefficients *c*_2,0_ and *c*_2,2_ and the equivalent sphere and cylinder power for a 4 mm pupil, with an artificial eye obtained by the HS-WFS and the DMD-WFS without binning.

	HS-WFS				DMD-WFS			
	*c*_2,0_[μm]	*c*_2,2_[μm]	Sphere[D]	Cyl.[D]	*c*_2,0_[μm]	*c*_2,2_[μm]	Sphere[D]	Cyl.[D]
a)	6.638	0.308	11.50	0.75	7.519	−0.024	13.02	0.00
b)	0.627	−4.867	1.08	−11.90	0.561	−6.511	0.97	−15.95
c)	6.270	−2.472	10.85	−6.05	7.084	−3.769	12.27	−9.23
